# The next generation of natural history collections

**DOI:** 10.1371/journal.pbio.2006125

**Published:** 2018-07-16

**Authors:** David E. Schindel, Joseph A. Cook

**Affiliations:** 1 Smithsonian Institution, Washington, D. C., United States of America; 2 Museum of Southwestern Biology, University of New Mexico, Albuquerque, New Mexico, United States of America

## Abstract

The last 50 years have witnessed rapid changes in the ways that natural history specimens are collected, preserved, analyzed, and documented. Those changes have produced unprecedented access to specimens, images, and data as well as impressive research results in organismal biology. The stage is now set for a new generation of collecting, preserving, analyzing, and integrating biological samples—a generation devoted to interdisciplinary research into complex biological interactions and processes. Next-generation collections may be essential for breakthrough research on the spread of infectious diseases, feeding Earth's growing population, adapting to climate change, and other grand research challenges. A decade-long investment in research collection infrastructure will be needed.

## Introduction

The financial challenges facing natural history museums have attracted significant media attention over many years [1]. In this article, we open a separate but related debate—the future of the collections of biological specimens and samples contained in these museums. As described below, the value and impacts of these scientific assets are beyond question. We suggest that the time is right to ask how we should build the next generation of collections in much the same way that astronomers and astrophysicists discuss and plan for future infrastructure needs of their disciplines. As our colleagues in those disciplines have demonstrated, all the major stakeholders need to participate in this planning process: researchers, research institutions, major research infrastructure (museums and other repositories, in this case), and funding agencies. Advances in the natural history collection community over the past decades have modernized the study of organisms. Here, we propose an expanded view of specimen collecting and the materials and data generated by these collections. This expanded view can enable generations of research on the complex and dynamic interactions among organisms, communities, and species.

## Recent advances

Biological collections have been undergoing a technological and cultural transformation over the past few decades. Accumulated over centuries in thousands of separate institutions (universities; private nonprofit organizations; local, state, and federal government agencies), three new trends began in the 1970s. First, institutions began to computerize their specimen catalogs, primarily to improve collection management and then later to increase the visibility of collections to potential users. Second, museums expanded the scope of the materials that are collected (e.g., frozen tissues, audio recordings, parasites) to take advantage of new technologies that can characterize individual organisms more fully (e.g., their genetics, isotopic content, internal anatomy, and behavior). This concept of the "extended specimen" is admirably developed for ornithological research in Webster [2]. "Holistic sampling" has built on the extended specimen concept by collecting, curating, and analyzing the ectoparasites, endoparasites, and microbiomes found on hosts and the pathogens they carry [3]. Third, the museum community has embraced and implemented a culture of data sharing that required development of data standards for taxonomic names, georeferences of collecting localities, and other data and metadata attached to specimens and data connectivity to associated data repositories (e.g., GenBank). These new practices spread rapidly in the United States with support from the National Science Foundation (NSF) and other funders, most recently through NSF's Advancing Digitization of Biological Collections (ADBC) Program. Parallel efforts are underway in the European Union, Australia, Canada, and elsewhere.

This evolution of biological collections from disconnected, stove-piped fiefdoms into an integrated global enterprise of samples and data has yielded significant dividends [4]. New uses and new users have emerged. Biobanks of frozen tissue have catalyzed an explosion in genomic studies. Specimen digitization and data sharing have led to the growth of data aggregators such as the Global Biodiversity Information Facility (GBIF) [5], VertNet [6], and iDigBio [7]. Web-accessible georeferenced specimen records through data aggregators have emerged as extraordinary resources for the study of changing distributions of species and populations over time [8–10]. Linking distributions with known characteristics of species has enabled niche modeling that predicts distributional shifts driven by environmental change or invasive species [11,12]. Online specimen catalogs now allow researchers to rapidly find specimens they need, greatly reducing the cost, effort, and delay associated with fieldwork. In some cases, online specimen-based digital images or public data sets of specimen traits or measurements are available. Resources such as GenBank for gene sequences, MorphBank for measurements, IsoBank for isotopic data [13], and MorphoSource for computed tomography (CT) scans [14] are creating opportunities to aggregate and analyze specimen data across populations, taxa, and regions while reducing cost, effort, and delays.

For the most part, these changes involve the management of specimens after they've been collected. Standards for the collection and preservation in the field have changed relatively little over centuries, with the exception of recording the global positioning system (GPS) location and preserving new types of samples for subsequent DNA and RNA analysis (e.g., tissues, gut contents, and parasites). That is, we have continued to collect and preserve specimens and we record the place, date, and time of the collecting event with a specific goal in mind (primarily research in taxonomy, evolutionary biology, or ecology).

## The next big thing

Complex biological interactions are of intense interest to a wide range of researchers, including those who make and use natural history collections as well as those in applied research linked to human health, food security, environmental quality, and even national security. At the same time, other disciplines are making and managing research collections that are rarely used for research in the natural history community. We believe that "Next-Generation Collections" (NGCs; see Box 1) will bridge this gap, thereby catalyzing decades of research that spans disciplines and connects fundamental and applied research. We foresee four initiatives needed to further establish NGCs as a central component of biological research infrastructure:

1. Promoting data standardization, digitization, data sharing and aggregation in ways that more directly connect natural history collections to other relevant disciplines (e.g., biomedicine, agriculture, veterinary science, cell culture and microbial collections, archaeology);
2. Expanding the concept of a "collecting event" to include multiple specimens of different species (e.g., the community) preserved at the same place and time so that biological processes of interest can be studied in greater detail. This holistic approach builds on the extended specimen sampling philosophy by collecting diverse specimens, samples and data types for a broader range of uses and users. Although holistic sampling involves higher operational costs, we believe that the potential returns on investment will justify the added expense;
3. Building the informatics capacity to connect collections and research in different disciplines, including all specimens and associated samples that result from a collecting event (even after being dispersed to different researchers and institutions), the institutions that maintain them, the researchers that generate new data on the specimens (e.g., genomes), and the images and trait data deposited in public repositories; and
4. Promoting and supporting interdisciplinary research that will build and use NGCs to unravel the mysteries of biological processes on scales from cells to ecosystems.

**Box 1. What makes NGCs different?**

**Fig 1:**
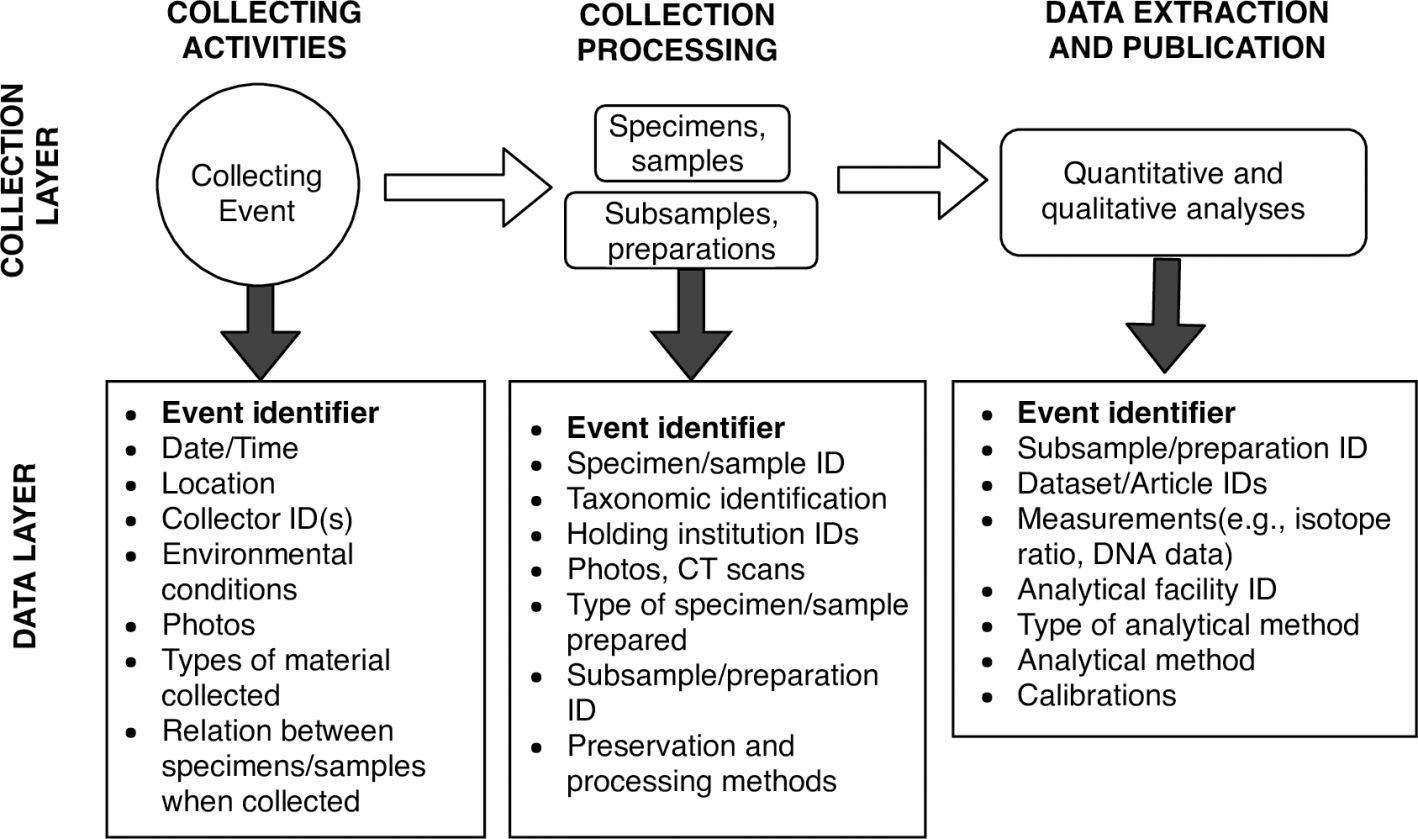
Collecting event cascade. Holistic sampling and extended specimens begin with a more comprehensive collecting event that captures multiple specimens, samples, and data about event context. These begin a cascade of subsamples, preparations, and diverse data records that may be located in different collections, institutions, data repositories, and publications. All these descendants and associated derivative data are linked to the original collecting event and, as a result, to each other.

Most natural history fieldwork today samples a restricted taxonomic group, such as a species of plant or animal, all the insects in a tree or caught in a trap, or a group of fish in the same family. NGCs would be expanded to encompass a wider range of the organisms and environmental samples. For example, holistic sampling could include an animal, all of its associated parasites and commensals, the surrounding plants and their associated fungi, and the underlying soil. Unlike most natural history specimens, items in a particular NGC may be distributed to specialists at different institutions and accessioned into different institutional collections. The data about these items and derived from them may be released in distributed public databases. Associated research findings may be published in articles in different disciplines. Using and building on these research findings will require durable informatics linkages among all the data derived from that original collecting event (see Fig 1).

### Looking forward

These examples of how these NGCs can be used for fundamental and applied research show how NGCs can become standard community practices (Box 2) and can benefit diverse stakeholders. Museum collections made over time could be revealing the dynamics of potential vectors, reservoirs of pathogens, sources of agricultural pests, and potential control agents. Historic samples are now being compared with newly collected specimens or clinical records to reveal changes in distribution, ecology, and transmission pathways [19–21].

**Box 2. Holistic sampling for infectious disease research**

**Fig 2:**
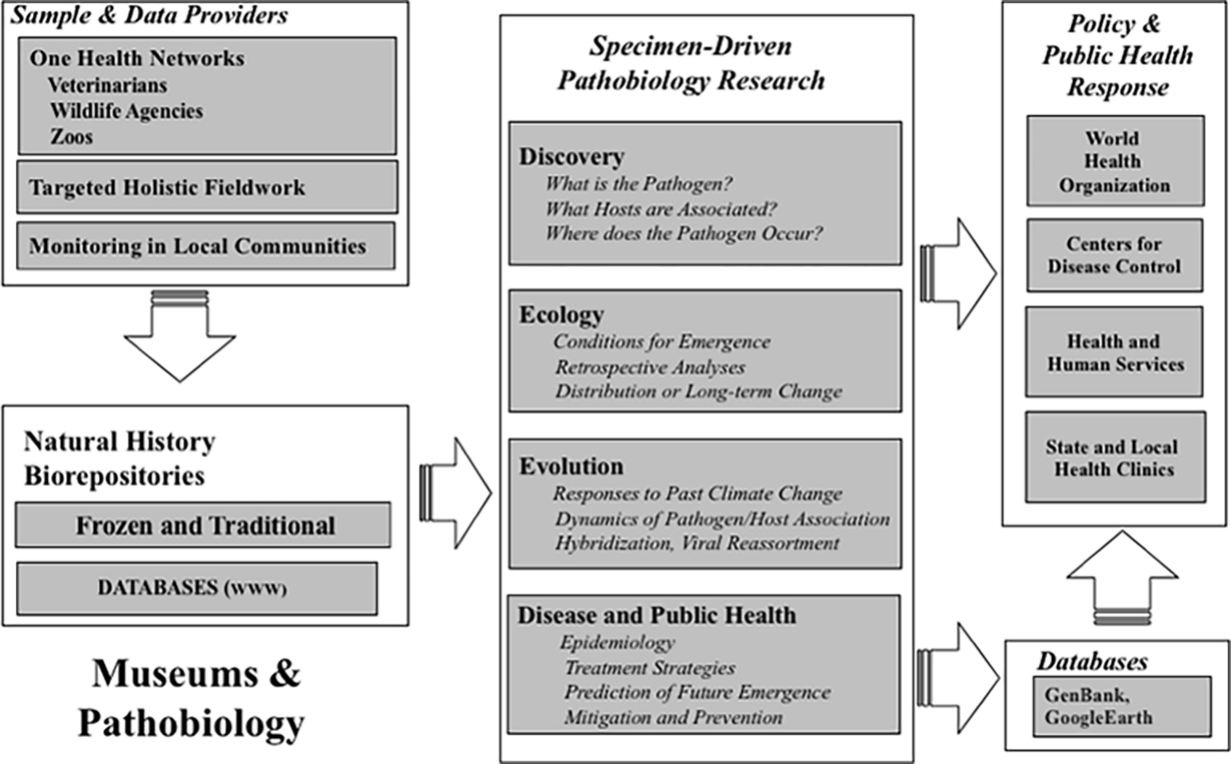
The role of natural history biorepositories in pathogen biology and mitigation. Host–parasite collections provide an exemplar of how museums can stimulate better coordination and participation in pathobiology across multiple institutions. Their roles range from sample providers to sample users (research) to informatics resources and contributing to the mitigation of public health crises. In this model, specimens are provided to natural history repositories by existing public health networks, fieldwork, and rural communities. Frozen and traditional collections become central to pathobiology research aimed at identifying pathogens, discovering zoonotic host associations, and delineating the potential spatial extent of the pathogen. Detailed questions about the pathogen ecology and evolutionary history can then be addressed to provide a framework for more effective public health response in increasingly dynamic environments. Relational web-accessible databases at museums facilitate complex linkages between all associated materials and allow careful tracking of all studies and their derived data (e.g., GenBank).

Pathobiology research increasingly relies on availability of frozen tissue archives in museums for comprehensive screening for diverse zoonotic pathogens. These archives have developed archival and database standards that ensure best practices in pathogen discovery, making them a major advance in rapid and rigorous assessment, prevention, and mitigation of emerging diseases [15]. Emerging pathogen questions can range from molecular identification of the pathogen to predictions of how changing environmental conditions will impact disease emergence in the future. It is therefore critical that all aspects of pathogen biology are integrated. For example, individual Q of a mammalian host (a specimen) is suspected of being a zoonotic host, and it corresponds to mammalian genome A with tissue-specific expression profiles (B, C, D), stable isotope signatures (E, F, G), with multiple associated endoparasites (R, S, T), ectoparasites (U, V, W), microbiomes (X, Y, Z), and so forth [3]. Preserving and connecting these diverse data streams helps to serve international initiatives on verification and data accessibility, transparency, and integration, which have been key challenges for pathogen biology and public health initiatives (see Fig 2).

Similarly, food security researchers (Box 3) are beginning to use herbarium specimens to understand how wild crop relatives respond to environmental stress, biological pests and pathogens, and invasive species in ways that can improve crops [21–25]. Unlike standard collecting protocols, however, more holistic sampling for these applications would cover all the species that participate in complex food web, predator–prey, and host–parasite relationships. Viewed generally, holistic sampling of communities is designed to capture species interactions, not just the characteristics of single species.

**Box 3. NGCs for integrated pest management**

**Fig 3:**
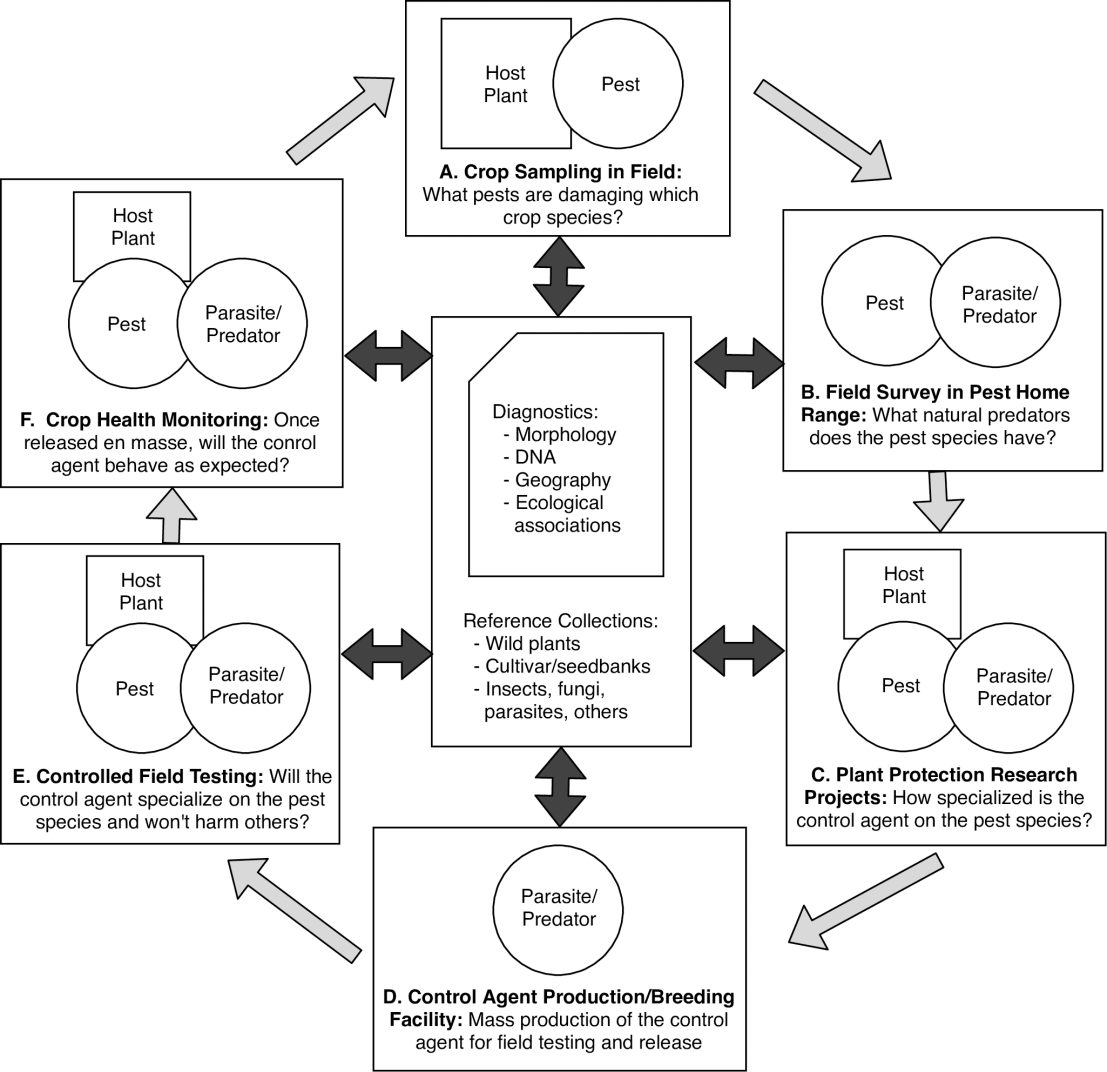
The role of biological collections in integrated pest management. The process of diagnosing an agricultural pest and then finding, testing, mass-producing, and releasing a control agent takes place in different settings and institutions. For the process to succeed, each participant in the process must be using the same host plants, pest species, and control agent species. Morphological and genetic comparisons with reference collections and databases can reveal the inadvertent introduction of look-alike or cryptic species into the process.

Climate change and international travel and trade are introducing species into new geographic regions. When new regions lack the natural population controls such as predators, introduced species can expand rapidly and threaten established species. Agricultural pests are a particularly important type of invasive species. Rather than relying on pesticides, governments and growers often try to introduce a "control agent"—a predator or parasite that will specialize on the invader but not harm crops and native species [16]. Fig 3 shows the Integrated Pest Management (IPM) process of seeking, identifying, and testing candidate control agents before they are mass produced for release. Testing is critical to ensure that the control agent will specialize only on the pest species. At each step in the process, there's a risk of using a "look-alike" or undescribed close relatives [17] instead of the correct host plant, pest species, or candidate control agent. IPM projects can fail as a result of misidentifications [18], wasting valuable time and resources while the pest remains unchecked. For this reason, host plants, pest species, and candidate control agents need to be carefully characterized morphologically and genetically and made available for comparison in reference collections.

## Conclusions

We believe that this new generation of collections and collections-based research can begin almost immediately. NSF's ADBC Program and earlier NSF initiatives began with planning workshops that led to implementation plans. A similar pathway for a major investment in NGCs in the US could lead to a multi-agency 10-year initiative shared among the National Science Foundation (NSF), United States Department of Agriculture (USDA), Department of the Interior (DOI), National Institutes of Health (NIH), Centers for Disease Control (CDC), and other agencies. This initiative could support a variety of integrative activities: interdisciplinary research symposia for planning new research programs that rely on NGCs; community workshops for development of interdisciplinary data standards, informatics tools, and data management infrastructure; and priority projects to build and digitize the first comprehensive collections. We believe that a ten-year investment in NGCs will move understanding of our dynamic living world to a new level.

## Abbreviations

ADBC: Advancing Digitization of Biological Collections
CDC: Centers for Disease Control
CT: computed tomography
DOI: Department of the Interior
GBIF: Global Biodiversity Information Facility
GPS: global positioning system
IPM: integrated pest management
NGC: Next-Generation Collection
NIH: National Institutes of Health
NSF: National Science Foundation
USDA: United States Department of Agriculture
